# Interactions Between Child Personality and Parenting in Relation to Child Well-Being: Support for Diathesis–Stress and Differential Susceptibility Patterns

**DOI:** 10.3389/fpsyg.2021.558224

**Published:** 2021-08-03

**Authors:** Svetlana V. Loginova, Helena R. Slobodskaya

**Affiliations:** Department of Child Development and Individual Differences, State Scientific Research Institute of Physiology and Basic Medicine, Novosibirsk, Russia

**Keywords:** diathesis-stress, differential susceptibility, person-by-environment interactions, child well-being, preschool age

## Abstract

It is well-recognized that the individual characteristics of children moderate the effects of developmental conditions on the well-being of a child. The majority of interactions follow a diathesis–stress pattern; there is also evidence for differential susceptibility and vantage sensitivity models. The present study aimed to examine interactions between parenting and child personality in relation to the well-being of a Russian child and to evaluate the models for moderated relationships. Participants were primary caregivers of 370 children aged 2–7 years. Moderation effects were examined using hierarchical multiple regression and bivariate linear models. In order to differentiate between the models of environmental sensitivity, the analysis of regions of significance was used. Consistent with a diathesis–stress framework, the results revealed that among children low in conscientiousness and high in activity, punitive parenting was a risk factor for externalizing problems; among introverted and fearful children, punitive parenting was a risk factor for internalizing problems. Positive parenting/involvement was a protective factor for internalizing behavior, only for children low in openness. The findings also demonstrate the following evidence for the differential susceptibility model: children low in Beta higher-order personality trait (also known as plasticity or personal growth) showed more total problems when faced with low positive parenting, but fewer problems when experiencing high-quality parenting.

## Introduction

Decades of research have proved that the individual characteristics of children and environmental conditions, especially parenting, make important contributions to the well-being of a child (Leve et al., 2005; Davidov and Grusec, 2006; Clerkin et al., 2007; De Pauw and Mervielde, 2010). It has been demonstrated that temperament and personality constructs, which have been seen as biologically based variations in emotion and regulation, are related to important developmental outcomes (Caspi and Shiner, 2008; Rothbart, 2011). Temperament and personality research traditions were largely independent for many years; however, in the current thinking, temperament and personality traits are more alike than different (Shiner and DeYoung, 2013). Therefore, in the current study, we use the terms temperament and personality interchangeably.

Structural research has demonstrated that many temperament and personality models can be integrated into a single hierarchical model (Markon et al., 2005). The Big Five traits, such as extraversion, neuroticism, agreeableness, conscientiousness, and openness, subsume more specific, mid-level traits and represent the foundational level of superordinate trait models (Slobodskaya, 2021). The higher-order factors of the Big Five, Alpha comprising agreeableness, conscientiousness, and neuroticism (reversed), and Beta comprising extraversion and openness, have been consistently recovered in studies with children, adolescents, and adults (Slobodskaya, 2011; Shiner and DeYoung, 2013). The two higher-order factors embrace three biologically based temperamental dimensions of positive emotionality, negative emotionality, and effortful control (Rothbart, 2011). Alpha, also known as stability or successful socialization, appears to reflect stable functioning in the emotional, motivational, and social domains, whereas Beta, also known as plasticity or personal growth, appears to reflect the tendency to explore behaviorally and cognitively (Digman, 1997; DeYoung, 2006).

Neuroticism and its components, such as fearfulness and anxiety are the major predictors of internalizing problems, whereas extraversion, low agreeableness, and conscientiousness are the main predictors of externalizing problems (De Pauw and Mervielde, 2010). It has also been shown that behavioral and affective aspects of parenting, such as monitoring and supervision, harsh punishment, inconsistent discipline, positivity, involvement, and responsiveness, are strong predictors of social, emotional, and behavioral adjustment of children (Leve et al., 2005; Davidov and Grusec, 2006; Clerkin et al., 2007). Although the effect of parenting on child well-being ranges from small to moderate, many randomized controlled trials have demonstrated that changing the parenting of young children led to a reduction in child behavioral problems by comparison with an untreated control group (Thomas and Zimmer-Gembeck, 2007).

There is extensive evidence that a given parenting practice may have different effects on different children, depending on age, sex, genetic, and temperament/personality characteristics (Chess and Thomas, 1991; Kiff et al., 2011; Slobodskaya et al., 2013; Slagt et al., 2016b; Stoltz et al., 2017). These findings also demonstrate that children differ in the degree and even in the kind of their response to environmental influences, especially the quality of parenting. The bioecological theory of Urie Bronfenbrenner underlies several models that posit how individual characteristics of children lead to variation in the sensitivity to the environmental conditions (Bronfenbrenner and Morris, 1998). The diathesis–stress or dual risk model proposes that vulnerable children are the most affected by environmental risk factors, such that temperament/personality can exacerbate the adverse effects of poor parenting. Based on the evolutionary-biological analysis of human development, Jay Belsky proposed the differential susceptibility model, which posits that some children are not just more vulnerable to the negative effects of maladaptive parenting but also benefit more from supportive and positive parenting (Belsky and Pluess, 2009). The vantage sensitivity model suggests that some children can benefit more from positive environmental experiences to which they are exposed (Pluess and Belsky, 2013).

The differential susceptibility framework includes both the diathesis–stress and the vantage sensitivity components. However, it is important to remember that these three types of environmental sensitivity may describe different individuals. That is, some individuals are relatively more vulnerable to the adverse effects of negative environments and do not differ from others in advantaged contexts, in line with the diathesis–stress model. Others are most likely to benefit from positive environmental experiences but do not differ from others in disadvantaged contexts, displaying vantage sensitivity (Pluess and Belsky, 2013). Still others are both relatively more affected by adverse environments and more likely to benefit from positive experiences, displaying differential susceptibility.

Most of the empirical findings have revealed a diathesis–stress pattern. For example, in a Dutch study, the interaction of parental responsiveness with impulsivity and low effortful control predicted externalizing problems 2 years later, and the interaction of harsh parenting with negative emotionality predicted low prosocial behavior (Slagt et al., 2016b). It has been shown that negative affectivity strengthens the relation of the parenting quality to the externalizing behavior of the children (Stoltz et al., 2017). A Russian study provided evidence that disharmonious family environment, including harsh parental discipline, poor parent–child relationships, alcohol abuse, domestic violence, and lack of cohesion and social support, strengthens the relation of neuroticism and extraversion and their mid-level traits to children's internalizing problems, and the relation of extraversion, sociability, and antagonism to externalizing problems (Slobodskaya et al., 2013). A review summarizing the existing findings of temperament × parenting interactions concluded that parental corporal punishment, overprotection, and intrusiveness predicted mental health problems for children high in irritability and negative emotionality; inconsistent discipline was related to depression and behavioral problems for highly impulsive children; and parental rejection predicted problem behavior for children low in positive emotionality (Kiff et al., 2011).

Some studies have supported the differential susceptibility model, showing that some temperamental characteristics served as plasticity factors (Pluess and Belsky, 2010). Longitudinal studies have shown that children with difficult temperament and high in negative emotionality had more externalizing problems than other children if they experienced harsh parenting, but fewer problems if they experienced high quality and sensitive parenting (Slagt et al., 2016a). A study from the United States has shown that compared to low-plasticity peers, children high in biobehavioral plasticity had more internalizing problems at ages 5.5–12 years if there was parental discord at age 4.5, but fewer problems when the relationship between their parents was harmonious (Brock et al., 2017). A recent study found that in the context of lower quality guided learning, 5-year-old children high in surgency showed lower levels of executive function skills than their low-surgency peers, but in the context of higher quality guided learning, they showed higher levels of executive function than children low in surgency (Sour et al., 2019). In another study, boys high in positive emotionality were especially susceptible to the effects of chronic interpersonal stress on the levels of social anxiety and depression, for better or for worse (Griffith et al., 2020).

Researchers generally agree that when examining environmental sensitivity, it is important to consider a full range of environments, both positive and negative, and a full range of developmental outcomes, including mental health problems and competent functioning (Belsky and Pluess, 2009; Slagt et al., 2016a). A broad coverage of the study enables investigators to reveal the difference between the three models. Support for the differential susceptibility hypothesis was initially found in studies examining interactions between temperament and environmental conditions in infancy and toddlerhood (Belsky and Pluess, 2009). There is some evidence supporting the differential susceptibility model in later childhood (Slagt et al., 2016b; Brock et al., 2017; Griffith et al., 2020). However, it is not yet clear whether the effects of rearing on the development of preschool children are similarly moderated by their individual characteristics and what traits would be the markers of differential susceptibility at different developmental stages (Slagt et al., 2016a).

Belsky and Pluess (2009) suggested that difficult temperament may reflect a highly sensitive nervous system on which environmental conditions and experience register especially strongly, both for better and for worse. However, they have also noted that the findings could be an artifact of the disproportionate attention that researchers paid to difficult temperament (Pluess and Belsky, 2010). Therefore, it is important to explore other potential behavioral markers of differential susceptibility rather than to replicate one such marker. It is also important to recognize that some plasticity markers may confer differential susceptibility, that is, both risk and vantage sensitivity, whereas others may confer only vulnerability to risk, and still others may confer only vantage sensitivity (Pluess and Belsky, 2013). Previous studies have focused mainly on genotypes or temperament traits (Belsky and Pluess, 2009; Kiff et al., 2011; Slagt et al., 2016a; Tung et al., 2019), whereas our study explores the role of child personality traits as potential moderators of parenting influence.

Recent research shows that connections between child personality and well-being can occur at different levels of trait hierarchy, so it is important to consider the role of higher- and lower-order traits besides the Big Five (Slobodskaya et al., 2013). There is some evidence suggesting that some specific traits from the same domain may exert different effects on problem behaviors. For example, in the neuroticism domain, fearfulness is closely linked to internalizing problems, whereas irritability and anger-proneness are specifically related to externalizing problems (Caspi and Shiner, 2008; De Pauw and Mervielde, 2010). It is also important that in some cases, the higher-order trait may be more closely related to child well-being than the lower-order traits from this domain, while in the others, the effect of the lower-order trait may be stronger than that of the higher-order trait.

Thus, in a Russian study, neuroticism was more strongly associated (positively) with internalizing problems, and conscientiousness was more strongly associated (negatively) with externalizing problems, whereas lower-order traits showed weaker relationships with problem behaviors (Slobodskaya and Akhmetova, 2010). At the same time, lower-order traits of intelligence and negative affect were more closely related to externalizing problems than their superordinate traits. While most studies on the relations of parenting and child personality to problem behaviors and well-being have focused on the Big Five traits, there is considerable evidence on the neurobiological developmental processes associated with the two higher-order factors of the Big Five and the three temperament factors, such as positive emotionality, negative emotionality, and effortful control (Rothbart, 2011; Shiner and DeYoung, 2013). Considering different levels of trait hierarchy can provide useful information about common and specific pathways and suggest possible developmental mechanisms and processes for child well-being.

The present study aimed to examine whether children vary in their sensitivity to parenting depending on their personality, and if so, which model might be considered the best for describing this sensitivity pattern. We considered three levels of the hierarchical personality structure: two higher-order factors Alpha and Beta, the Big Five, and mid-level traits. The results of longitudinal and cross-sectional studies on the type of interactions between parenting and individual differences of children are mixed (Kiff et al., 2011; Slagt et al., 2016a). Therefore, we do not formulate hypotheses concerning the direction and type of moderation, that is, whether differential susceptibility or diathesis–stress or vantage sensitivity effects will be domain-specific or domain-general with regard to child outcomes.

## Materials and Methods

### Participants

A community sample consisted of parents of preschool children from Siberia. Most of the families were living in Novosibirsk, the third largest city in Russia; 20% were from a thereabout town, and 10% were from villages. There were reports of 370 children from primary caregivers (53% males) aged 2–7 years (M = 5.1; SD = 1.3 years). Children aged from 3 to 6 years constituted 88.1% of the sample and were approximately equally represented within each year group:

$$\begin{array}{l} \chi^{2}=4.14,\text{df}=3,\text{p}=\;0.247. \end{array}$$

Most of the children (92%) were rated by mothers, 7% by fathers, and 1% by both parents together. About 89% of the children lived with two biological parents, 8% with a single parent, and 3% with the mother and the stepfather. Compared to the general population of Novosibirsk region, the sample of this study had a similar proportion of participants with a technical school education (25% of mothers and 38% of fathers) and more participants with a university education (70% of mothers and 53% of fathers). For occupation, the parents ranged from unskilled (19% of mothers and 29% of fathers) or manual workers (30% of mothers and 37% of fathers) to specialists and administrative staff (30% of mothers and 35% of fathers); 21% of mothers and 2% of fathers were unemployed or students.

### Procedure

The sampling aimed to collect data from varied socioeconomic backgrounds. Participants of the study were recruited through several methods. Caregivers were approached *via* 10 non-selective public kindergartens (*N* = 264, 71% of the total sample), sports club for children (*N* = 17, 5%), and also by research assistants and students who contacted parents with a preschool-aged child (*N* = 84, 23%); if the caregivers agreed to participate, they received the questionnaires, completed, and returned them to the investigators.

### Measures

#### Personality

The Inventory of Child Individual Differences, short version (ICID-S, Slobodskaya and Zupancic, 2010) is a culturally and age-decentered parent-report measure designed to assess the personality of the child. The ICID-S consists of 62 items to measure 15 mid-level traits comprising five higher-order factors. Extraversion includes sociability (likes to be with people), positive emotions (sweet, loving), and activity level (energetic, active physically). Neuroticism comprises negative affect (irritable, quick-tempered), shyness (socially reticent), and fearfulness (insecure, lacks confidence). Openness includes being open to experience (imagination, tendency to explore) and intelligence (good memory and thinking abilities). Conscientiousness comprises being compliant (cooperative in response to authority), organized (orderly and tidy), achievement orientation (self-discipline and focus on goal attainment), and reversed distractible (poor concentration, short attention span). Disagreeableness includes strong will (bossy, self-assertive), antagonism (confrontational behavior), and reversed considerate (concerned about others).

The Russian version of the ICID has shown good reliability and full measurement invariance of the five-factor structure across informants, genders, and ages 2–15 (Knyazev et al., 2008). Another study confirmed full measurement invariance of the two-factor structure of the ICID Big Five across informants aged from 3 to 17 in the Russian sample (Slobodskaya, 2011). In the present study, scores for the mid-level scales and the Big Five were created by summing the relevant items. The internal reliabilities for the ICID-S mid-level scales (Cronbach's α coefficients) ranged from 0.60 to 0.86, with a mean of 0.77; the internal reliabilities for the Big Five ranged from 0.84 to 0.90, with a mean of 0.87. Alpha and Beta higher-order factors were estimated by regression-based factor scores obtained from the exploratory factor analysis of the ICID-S Big Five scales (Slobodskaya, 2011).

#### Parenting Practices

The Alabama Parenting Questionnaire—Preschool Revision (APQ-PR, Clerkin et al., 2007) is a measure of empirically identified positive and negative parenting characteristics for preschool-age children. The Russian version of the APQ-PR has been validated in a community sample, showing support for the original three-factor structure, good internal consistency, discriminant, and criterion validity of the scales (Loginova et al., 2016). A 7-item positive parenting/involvement scale (α = 0.66) includes four items on the positive parenting (e.g., “You hug or kiss your child when he/she has done something well”) and three items on parental involvement (e.g., “You volunteer to help with special activities that your child is involved in”). The inconsistent parenting scale (α = 0.48) includes four items (e.g., “The punishment you give your child depends on your mood”). A punitive parenting scale (α = 0.84), includes the following six items: “You use time out (make him/her sit or stand in the corner) as a punishment;” “You yell or scream at your child when he/she has done something wrong;” “You ignore your child when he/she is misbehaving;” and three items on corporal punishment (e.g., “You slap your child when he/she has done something wrong”). Each of the items was scored from 1 (never) to 5 (always). We also used a 3-item corporal punishment subscale (α = 0.76) for additional analyses.

#### Well-Being

The strengths and difficulties questionnaire (SDQ, Goodman, 2001) is a 25-item measure of the mental aspects of child well-being covering prosocial behavior, common behavioral and emotional difficulties, and the impact of problems on everyday functioning of the child (Goodman and Goodman, 2009). Community studies provided evidence for a three-factor structure of the SDQ, with the 5-item prosocial behavior scale and the two 10-item scales, internalizing and externalizing (Goodman et al., 2010). The 20-item total difficulties scale has been found to be a psychometrically sound general measure of child mental health (Goodman et al., 2010) and well-being in studies around the world (Claessens and Chen, 2013; Hoosen et al., 2018). The Russian version of the SDQ has been validated in a stratified random sample, supporting its reliability and validity (Goodman et al., 2005). In the present study, Cronbach's alphas were 0.67 for prosocial behavior, 0.71 for externalizing problems, 0.60 for internalizing problems, 0.71 for total difficulties, and 0.73 for the impact scale.

### Data Analyses

Tests of the main and interactive effects of the child personality and the parenting practices on the child well-being were conducted using hierarchical multiple regression analyses. Outcomes were the SDQ scales for prosocial behavior, internalizing and externalizing problems, total difficulties, and impact. Following recommended procedures (Aiken and West, 1991), we used a hierarchical order of the entry of the predictor variables: covariates (sex and age of the child) were entered at the first step; the predictor variables (personality traits at one of the three levels of the hierarchical structure and parenting practices) were entered at the second step; two-way interactions of each parenting practice (positive parenting/involvement, inconsistent parenting, and punitive parenting) with child age and gender and each personality characteristic were entered at the third step.

There were three regression models at different levels of the personality hierarchy: Model 1 for Alpha and Beta superordinate factors, Model 2 for the Big Five, and Model 3 for the 15 mid-level traits. Additional analyses used the corporal punishment subscale instead of the punitive parenting. In order to have a common scale and to minimize multicollinearity, all variables were standardized before the interaction terms were calculated. To make the full use of the sample and not to cause potential bias over the listwise deletion, we used the EM algorithm in SPSS for missing data. Considering that tests of interactions generally have very low statistical power and a high risk of Type II errors (Whisman and McClelland, 2005; Blake and Gangestad, 2020), we used an unadjusted alpha level of 0.05. To reduce the probability that the results for interactions are due to the effect of multiple testing, we employed a three-stage selection procedure.

First, we tested multiple regression models. At the second stage, we tested significant interaction effects from the multiple regression analyses in separate models for the two moderating variables. Finally, we considered interactive effects of parenting which were reproduced at two or three levels of personality hierarchy. Following the recommendations by Roisman et al. (2012), we conducted the “regions of significance on X” (RoS on X) analysis that detects regions in the range of the moderator, where the effect of the predictor on the outcome variables is statistically significant. If the association between the moderator and the outcome is significant at one end of the predictor, there is evidence that the data support the diathesis–stress model. If this association is significant at both the low and high ends of the distribution of the predictor, there is evidence of differential susceptibility. The presentation of the results is organized by the outcome; personality traits are presented in the descending order by the higher-order factors of the Big Five, Alpha comprising agreeableness, conscientiousness, and neuroticism (reversed), and Beta comprising extraversion and openness.

## Results

### Preliminary Analysis

Bivariate associations of child personality traits and parenting practices with child well-being are presented in Table 1. Prosocial behavior was mostly related to the Alpha superordinate factor, agreeableness, conscientiousness, and neuroticism (reversed). With regard to parenting practices, prosocial behavior was positively associated with positive parenting/involvement and, to a lesser degree, negatively associated with punitive parenting. Externalizing problems were negatively associated with the Alpha domain, and were positively associated with Beta, extraversion and dysfunctional parenting practices (corporal punishment, punitive and inconsistent parenting). Internalizing problems and impact were negatively related to the Alpha superordinate factor, the agreeableness, conscientiousness, and openness domains, and positive parenting practices. They were also positively related to the neuroticism domain, inconsistent parenting, and corporal punishment. Internalizing problems were also negatively associated with the Beta higher-order factor and the extraversion domain. The impact scale was positively linked to punitive parenting.

**Table 1:** Correlations of child personality and parenting practices with child well-being.

**Measure**	**Prosocial behavior**	**Externalizing**	**Internalizing**	**Total difficulties**	**Impact**
**Child personality**					
**Alpha**	0.41^***^	−0.63^***^	−0.33^***^	−0.63^***^	−0.39^***^
**Disagreeableness**	−0.43^***^	0.49^***^	0.28^***^	0.50^***^	0.29^***^
Strong willed	−0.21^***^	0.48^***^	0.22^***^	0.46^***^	0.21^***^
Antagonism	−0.31^***^	0.45^***^	0.24^***^	0.45^***^	0.26^***^
Considerate	0.51^***^	−0.21^***^	−0.20^***^	−0.26^***^	−0.18^***^
**Conscientiousness**	0.36^***^	−0.62^***^	−0.25^***^	−0.57^***^	−0.38^***^
Achievement	0.35^***^	−0.45^***^	−0.20^***^	−0.43^***^	−0.28^***^
Organized	0.26^***^	−0.54^***^	−0.22^***^	−0.50^***^	−0.37^***^
Compliant	0.35^***^	−0.50^***^	−0.21^***^	−0.47^***^	−0.27^***^
Distractible	−0.22^***^	0.51^***^	0.18^***^	0.46^***^	0.31^***^
**Neuroticism**	−0.22^***^	0.18^***^	0.47^***^	0.39^***^	0.34^***^
Fearful	−0.12^*^	0.08	0.38^***^	0.27^***^	0.28^***^
Shy	−0.16^**^	−0.04	0.38^***^	0.20^***^	0.20^***^
Negative affect	−0.24^***^	0.48^***^	0.32^***^	0.51^***^	0.34^***^
**Beta**	0.11*	0.24^***^	−0.22^***^	**0.04**	**−0.09**
**Extraversion**	0.20^***^	0.17^***^	−0.27^***^	**−0.04**	−0.12*
Activity	0.06	0.32^***^	−0.13^*^	0.15^**^	−0.05
Sociable	0.21^***^	0.18^***^	−0.29^***^	−0.04	−0.13^*^
Positive emotions	0.21^***^	−0.11^*^	−0.24^***^	−0.21^***^	−0.08
**Openness**	0.18^***^	**−0.03**	−0.18^***^	−0.12*	−0.16**
Open to Experience	0.17^***^	0.05	−0.14^**^	−0.04	−0.11^*^
Intelligent	0.16^**^	−0.12^*^	−0.20^***^	−0.20^***^	−0.20^***^
***Parenting***					
Positive parenting	0.23^***^	−0.04	−0.11^*^	−0.09	−0.15^**^
Inconsistent parenting	−0.10	0.27^***^	0.16^**^	0.27^***^	0.13^*^
Punitive parenting	−0.15^**^	0.28^***^	0.08	0.24^***^	0.17^***^
Corporal punishment	−0.11^*^	0.30^***^	0.14^**^	0.29^***^	0.13^*^

* *p < 0.05;*

** *p < 0.01;*

*** *p < 0.001*.

The main effects of child personality traits and parenting practices on child well-being are presented in Table 2. Child age and gender did not contribute significantly to internalizing problems and accounted for around 2% of the variance in other well-being variables. Parents rated older children higher than younger ones on prosocial behavior and impact and lower on externalizing problems. Male gender was a risk factor for externalizing problems and total difficulties. Child personality traits at different levels of the hierarchy, together with parenting, explained 21–35% of the variance in prosocial behavior, 47–59% in externalizing problems, 16–30% in internalizing problems, 41–50% in total difficulties, and 20–26% in impact scores. Alpha, agreeableness, the mid-level trait of considerate, and positive parenting/involvement made significant contributions to prosocial behavior. Alpha, conscientiousness, and its mid-level traits of organized and low distractibility were protective factors for externalizing problems, whereas Beta, extraversion, its mid-level trait of activity, and inconsistent parenting were the risk factors. Both higher-order traits were protective factors for internalizing problems, whereas neuroticism and fearfulness were the risk factors. Alpha and conscientiousness were the protective factors and neuroticism was the risk factor for total difficulties and impact scores. Inconsistent parenting also contributed to total difficulties. The openness traits and punitive parenting did not make a significant contribution to well-being variables when other personalities and parenting variables were taken into account. However, additional analyses showed that corporal punishment made significant contributions to externalizing problems.

**Table 2:** Main effects of child personality and parenting practices on child well-being.

**Predictors**	**Model**	**Prosocial behavior**	**Externalizing**	**Internalizing**	**Total difficulties**	**Impact**
Step 1: R, R^2^,%		(0.13; 1.7)	(0.15; 2.4)	(0.07; 0.5)	(0.14; 2.0)	(0.15; 2.2)
Age		0.12^*^	−0.11^*^	0.01	−0.08	0.14^**^
Gender		0.04	−0.10^*^	−0.07	−0.11^*^	−0.04
Step 2: ΔR, Δ*R*^2^, %	M1	(0.46; 21.5)	(0.69; 47.2)	(0.40; 16.3)	(0.64; 40.9)	(0.45; 20.1)
	M2	(0.49; 23.8)	(0.74; 54.0)	(0.49; 23.7)	(0.66; 43.7)	(0.48; 23.1)
	M3	(0.59; 35.0)	(0.77; 59.2)	(0.55; 30.0)	(0.71; 50.4)	(0.51; 26.1)
**Alpha**	**M1**	0.37^***^	−0.58^***^	−0.31^***^	−0.58^***^	−0.37^***^
**Disagreeableness**	**M2**	−0.32^***^	0.16**	**−0.03**	**0.09**	**−0.04**
Strong willed	M3	−0.02	0.13^**^	0.09	0.14^*^	0.01
Antagonism	M3	0.00	0.01	−0.16^*^	−0.08	−0.02
Considerate	M3	0.55^***^	0.04	0.09	0.08	0.02
**Conscientiousness**	**M2**	0.15*	−0.58^***^	**−0.09**	−0.46^***^	−0.30^***^
Achievement	M3	0.11	−0.18^**^	−0.06	−0.16^*^	−0.03
Organized	M3	0.01	−0.19^***^	−0.05	−0.17^**^	−0.23^***^
Distractible	M3	−0.07	0.21^***^	−0.05	0.11^*^	0.05
**Neuroticism**	**M2**	**0.06**	**0.04**	0.40^***^	0.26^***^	0.28^***^
Fearful	M3	−0.01	−0.06	0.24^***^	0.10	0.10
Shy	M3	0.07	−0.04	0.15^*^	0.05	0.04
Negative affect	M3	−0.06	0.14^*^	0.18^*^	0.20^***^	0.15^*^
**Beta**	**M1**	0.10*	0.23^***^	−0.21^***^	**0.04**	**−0.06**
**Extraversion**	**M2**	0.16*	0.30^***^	**−0.09**	0.16*	**0.10**
Activity	M3	−0.12	0.26^***^	0.16^*^	0.27^***^	0.06
Sociable	M3	0.20^**^	−0.07	−0.24^**^	−0.18^*^	−0.12
Positive emotions	M3	−0.16^*^	0.00	−0.14	−0.08	0.19^**^
Positive parenting	M1	0.16^***^	0.01	−0.04	−0.01	−0.08
	M2	0.16^***^	0.02	−0.05	−0.02	−0.09
	M3	0.13^**^	0.01	−0.03	−0.01	−0.11^*^
Inconsistent parenting	M1	−0.01	0.12^**^	0.09	0.14^***^	0.03
	M2	−0.02	0.12^**^	0.07	0.13^**^	0.01
	M3	−0.05	0.11^**^	0.06	0.10^*^	0.01
Punitive parenting	M1	−0.01	0.04	−0.04	0.01	0.06
	M2	0.00	0.03	−0.00	0.02	0.07
	M3	−0.02	0.04	−0.03	0.01	0.07
Corporal punishment	M1	0.01	0.10^*^	0.06	0.10^*^	0.03
	M2	0.02	0.08^*^	0.09	0.11^*^	0.04
	M3	0.01	0.11^**^	0.04	0.08	0.04

*Gender is coded girls = 1, boys = 0. M1: Personality superordinate factors and parenting; M2: Personality Big Five and parenting; M3: Personality 15 mid-level traits and parenting. Standardized regression coefficients are shown.*

* *p < 0.05;*

** *p < 0.01;*

*** *p < 0.001*.

### Interactive Effects of Personality and Family Factors on Child Well-Being

There were a total of 20 significant (*p* < 0.05) interactions: four effects involving a higher-order factor, seven effects involving the Big Five, and nine interactive effects involving mid-level traits. Interactions between child age or gender and parenting practices were not significant. Among parenting practices, there were no interactive effects involving inconsistent parenting or corporal punishment, whereas the effects of positive and punitive parenting on child well-being were moderated by child personality traits. Several moderating effects have emerged at three levels of the personality hierarchy, indicating the replicability of the interactive pattern.

### Prosocial Behavior

**Figure 1:**
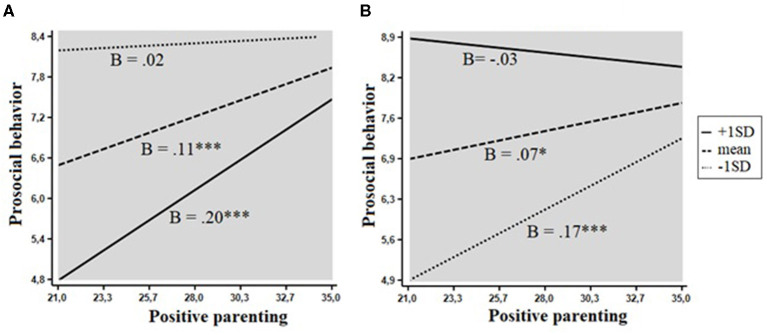
Interactions between personality and positive parenting in relation to prosocial behavior. The shaded area represents the region of significance. **(A)** Disagreeableness. **(B)** Considerate. *p < 0.05, ***p< 0.001.

The following two interactive effects on prosocial behavior were significant: positive parenting/involvement interacted with disagreeableness and its mid-level trait of considerate, *F*_(1, 351)_ = 6.30, *p* = 0.013 and *F*_(1, 351)_ = 10.60, *p* = 0.001, respectively. The evaluation of regions of significance revealed that both effects adhered to a diathesis–stress model (both RoS on X < 35.0). The contribution of positive parenting to prosocial behavior was larger in children with low levels of agreeableness and considerate, smaller in children with average levels of these personality traits, and non-significant in children with high levels of agreeableness and considerate (Figures 1A,B).

### Externalizing Problems

**Figure 2:**
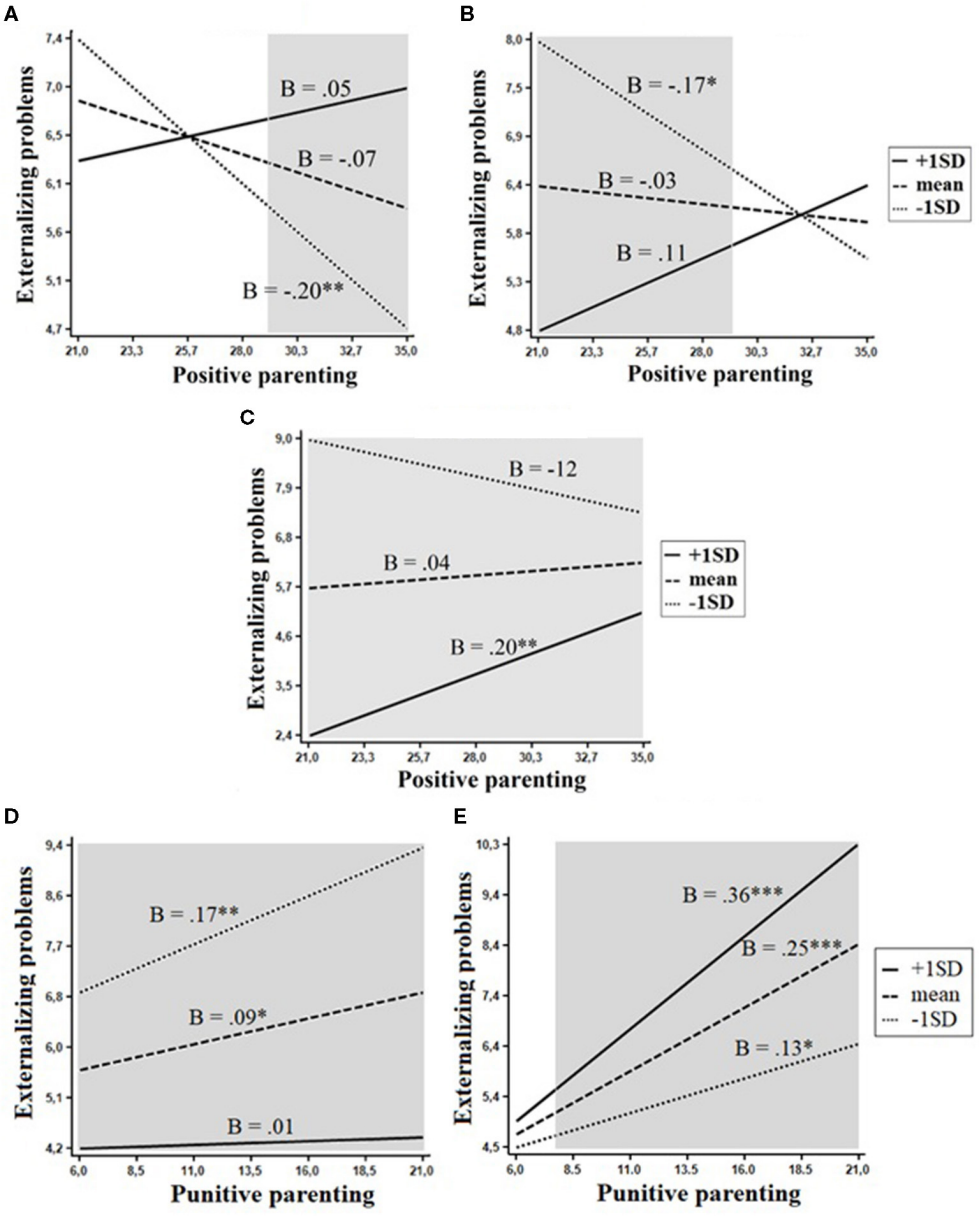
Interactions between personality and positive parenting in relation to externalizing problems. The shaded area represents the region of significance. **(A)** Beta. **(B)** Openness. **(C)** Organized. **(D)** Conscientiousness. **(E)** Activity. *p < 0.05, **p < 0.01, ***p < 0.001.

Five interactive effects on externalizing problems were confirmed in the models with the following two moderating variables: Positive parenting/involvement interacted with Beta, *F*_(1, 351)_ = 4.6, *p* = 0.034, openness, *F*_(1, 351)_ = 6.3, *p* = 0.012, and the trait of organized, *F*_(1, 351)_ = 10.8, *p* = 0.001; punitive parenting interacted with conscientiousness, *F*_(1, 350)_ = 3.9, *p* = 0.049, and the trait of activity, *F*_(1, 350)_ = 6.3, *p* = 0.013 (Figures 2A–E). Two of these effects, interactions of positive parenting with Beta and openness, emerged in the same personality domain. Although interaction plots shown in Figures 2A,B suggest the differential susceptibility model, the evaluation of regions of significance revealed that for both Beta and openness interactive effects of positive parenting adhered to a diathesis–stress model. Positive parenting/involvement was a protective factor for externalizing problems only among children low in Beta (RoS on X > 29.15) and openness (RoS on X <29.25). The association between punitive parenting and externalizing problems was the strongest in children low in conscientiousness, weaker in children with average levels of conscientiousness, and non-significant in highly conscientious children (Figure 2D). In a similar way, the strength of association between punitive parenting and externalizing problems increased with increasing levels of the trait of activity (Figure 2E).

### Internalizing Problems

**Figure 3:**
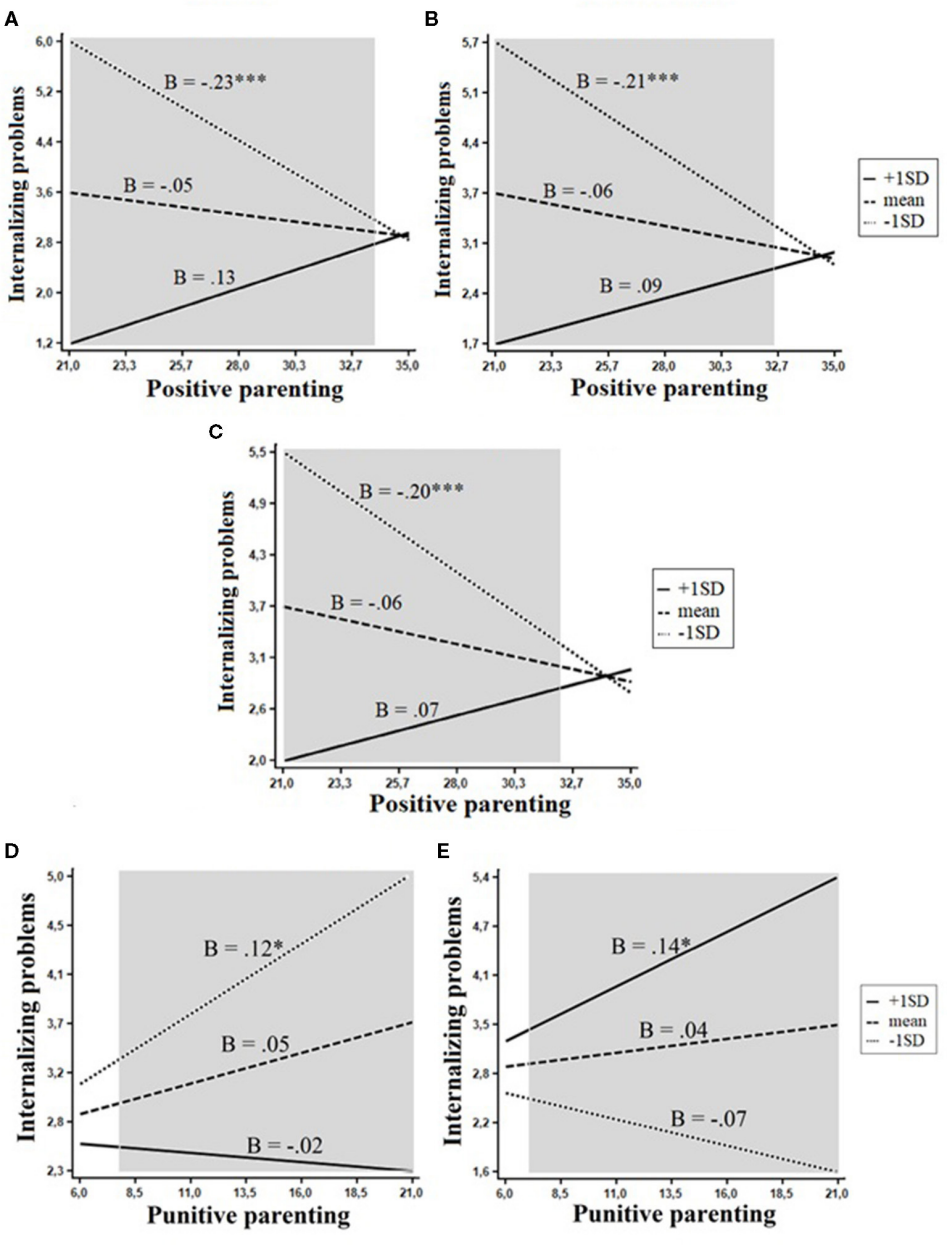
Interactions between personality and positive parenting in relation to internalizing problems. The shaded area represents the region of significance. **(A)** Beta. **(B)** Openness. **(C)** Openness to experience. **(D)** Extraversion. **(E)** Fearful. *p < 0.05, ***p < 0.001.

Five interactive effects on internalizing problems were significant. Positive parenting/involvement interacted with the Beta superfactor, *F*_(1, 351)_ = 14.1, *p* < 0.001, openness, *F*_(1, 351)_ = 10.3, *p* < 0.001, and the mid-level trait of openness to experience, *F*_(1, 351)_ = 8.8, *p* < 0.01, whereas punitive parenting interacted with extraversion, *F*_(1, 350)_ = 3.04, *p* < 0.05 and the trait of fearful, *F*_(1, 351)_ = 6.2, *p* < 0.05. Three of these effects, interactions of positive parenting with Beta, openness, and the trait of openness to experience, emerged in the same personality domain. The evaluation of regions of significance supported the diathesis–stress model: RoS on X < 33.05 for Beta, RoS on X <32.60 for openness, and RoS on X <32.03 for the trait of openness to experience. Thus, positive parenting was a significant protective factor for internalizing problems only among children with low levels of Beta, openness, and openness to experience. Children with average levels of these traits had average levels of internalizing problems, whereas children with high levels of Beta, openness, and the trait of openness to experience had relatively lower levels of internalizing, regardless of the level of positive parenting they received (Figures 3A–C). In a similar way, punitive parenting was a significant risk factor for internalizing problems only among children low in extraversion (Figure 3D) and among highly fearful children (Figure 3E).

### Total Difficulties

**Figure 4:**
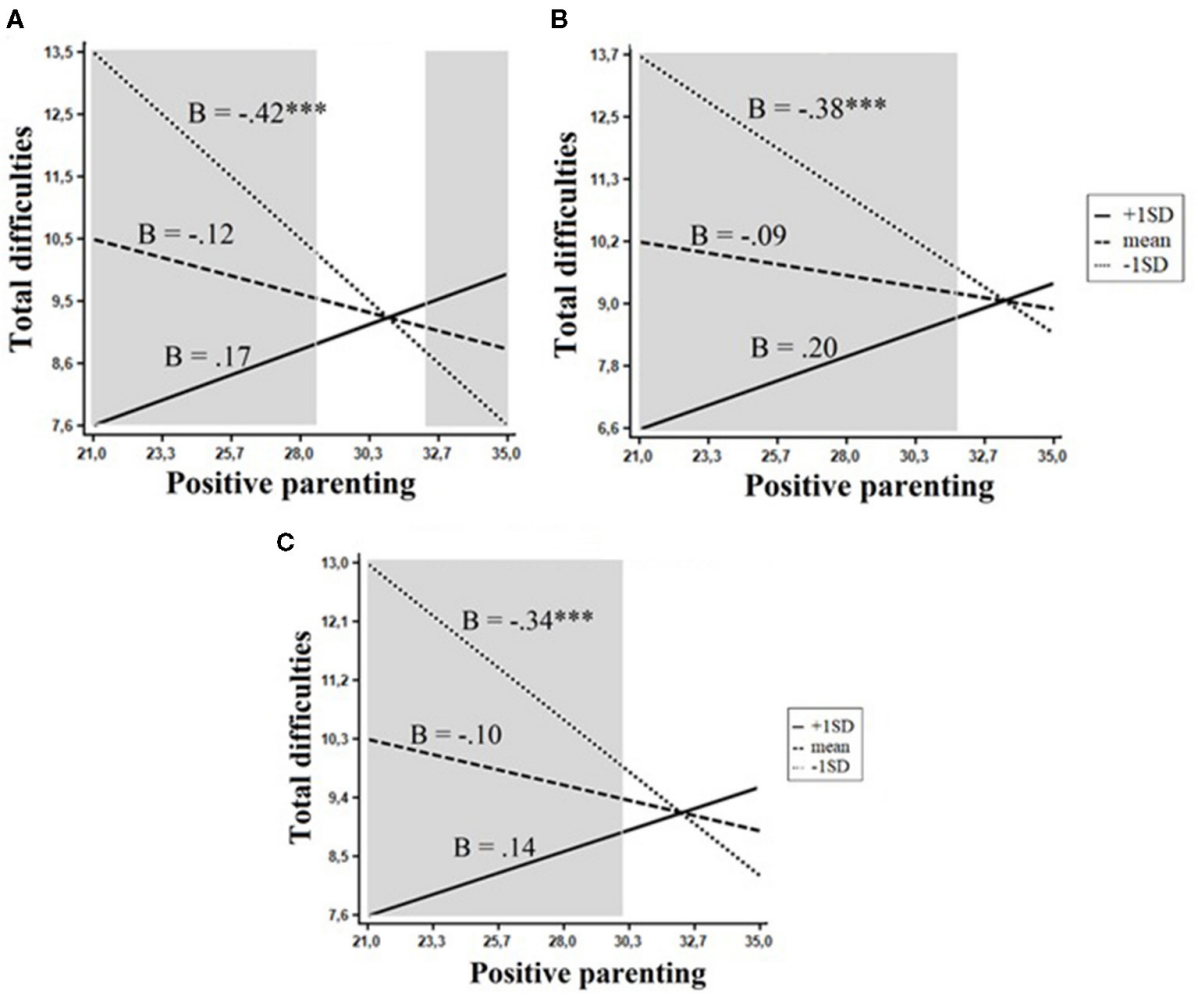
Interactions between personality and parenting practices in relation to total difficulties. The shaded area represents the region of significance. **(A)** Beta. **(B)** Openness. **(C)** Openness to experience. ***p < 0.001.

Three interactive effects on total difficulties were significant. Positive parenting/involvement interacted with Beta superfactor, *F*_(1, 351)_ = 11.9, *p* < 0.001, openness, *F*_(1, 351)_ = 12.2, *p* < 0.001, and the trait of openness to experience, *F*_(1, 351)_ = 9.2, *p* < 0.01. All these interactions emerged in the same personality domain. The evaluation of regions of significance revealed that the interaction of positive parenting and Beta was consistent with a differential susceptibility model: The lower and upper bounds of RoS on X were found to be 28.50 and 32.51, respectively. This means that when exposed to low positive parenting and involvement (RoS on X < 28.50), children low in Beta scored significantly higher on the total difficulties than children with high and average levels of Beta, whereas with high-quality parenting (RoS on X > 32.51); these children showed significantly fewer problems than children high and average in Beta (Figure 4A). The interactions of positive parenting with openness and its mid-level trait of openness to experience adhered to a diathesis–stress model (Figures 4B,C): Positive parenting was significantly and negatively associated with total difficulties only among children with low levels of these traits (RoS on X <31.73 and RoS on X <29.99, respectively).

### Impact of Problems

**Figure 5:**
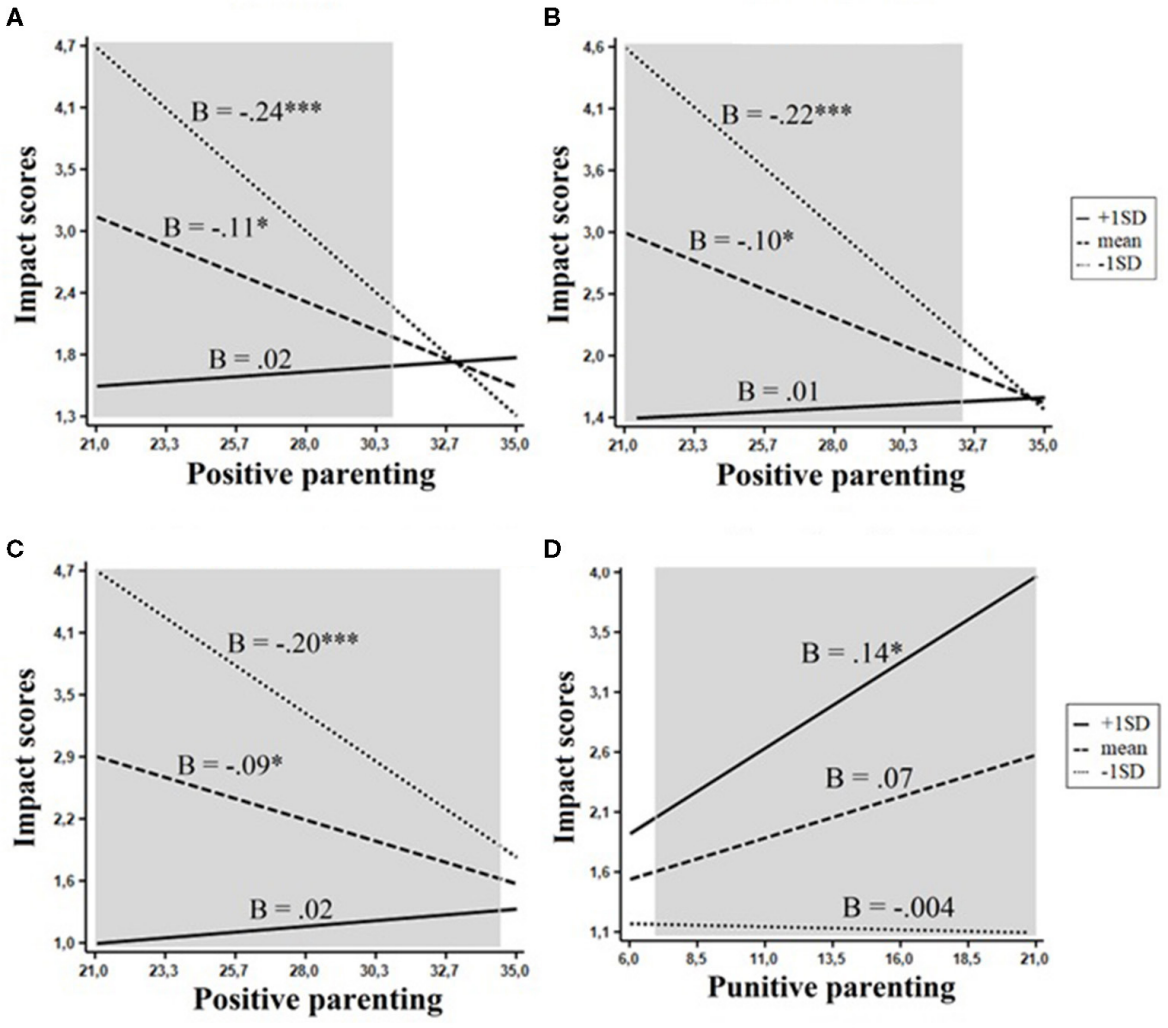
Interactions between personality and parenting practices in relation to impact scores. The shaded area represents the region of significance. **(A)** Beta. **(B)** Openness. **(C)** Achievement orientation. **(D)** Negative affect. *p < 0.05, ***p < 0.001.

Four interactive effects on the impact of problems were significant. Positive parenting/involvement interacted with the Beta superfactor, *F*_(1, 350)_ = 7.7, *p* < 0.01, openness, *F*_(1, 350)_ = 6.6, *p* < 0.01, and the trait of achievement orientation, *F*_(1, 349)_ = 7.4 *p* < 0.01, whereas punitive parenting interacted with the mid-level trait of negative affect, *F*_(1, 348)_ = 3.4, *p* < 0.05. Two of these effects, interactions of positive parenting with Beta and openness, emerged in the same personality domain. The evaluation of regions of significance revealed that both effects adhered to a diathesis–stress model. The protective effect of positive parenting on the impact of problems was largest in children with low levels of Beta and openness, smaller in children with average levels of these personality traits, and non-significant in children with high levels of Beta and openness (RoS on X <30.91 and RoS on X <32.35, respectively, Figures 5A,B). In a similar way, the negative association between positive parenting and the impact of problems was the strongest in children with relatively low levels of achievement orientation, weaker in children with average levels of achievement orientation, and non-significant in highly achievement-oriented children (Figure 5C). Punitive parenting was associated with the impact of problems only among children high in negative affect (RoS on X > 6.97, Figure 5D).

## Discussion

In the present study, we aimed to determine the main and interactive effects of parenting and child personality on the well-being of Russian preschool children. Importantly, the measures of well-being included not only negative but also the positive developmental outcomes, such as prosocial behavior, and beneficial environments, such as positive parenting/involvement. The results showed that parenting and children's personality traits are not only associated with child well-being, but also interacted with each other in meaningful ways. This study also addressed the important issue of the type of interactive effects and the type of model that can best be used to explain this pattern of sensitivity.

### Direct Effects

Our findings suggest that after controlling for child sex and age, the links between child personality traits and well-being were quite strong, which is generally consistent with previous research (De Pauw and Mervielde, 2010; Slobodskaya and Akhmetova, 2010). Interestingly, neither openness nor its mid-level traits of being open to experience and intelligent contributed significantly to well-being measures once other personality traits and parenting have been accounted for. This result was unexpected because a previous Russian study provided evidence of the links between openness to experience and both kinds of problems of children and between intelligence and externalizing problems (Slobodskaya and Akhmetova, 2010). It should be noted that the biological underpinnings of openness are not clear and it seems quite difficult to assess in young children, possibly because openness does not become developmentally relevant until middle childhood or even adolescence (Donnellan and Robins, 2009).

Our findings also confirmed the relations between parenting practices and child well-being, although these associations were weaker compared to the relations between child personality traits and well-being. The role of positive parenting practices in child well-being (children's negative affect regulation, empathy, prosocial responding, and peer acceptance) was shown in studies of young children (Davidov and Grusec, 2006; Clerkin et al., 2007). The links between inconsistent parenting and externalizing problems in children confirm the well-established evidence from other cultures. Unexpectedly, punitive parenting did not contribute significantly to children's problem behavior when other parenting practices and child personality traits have been accounted for, whereas corporal punishment made a significant contribution to externalizing problems and total difficulties. One possible explanation is that in our study, the measure of punitive parenting included not only corporal punishment but also effective behavioral management techniques, such as ignoring and time-out. While the relations of corporal punishment to externalizing problems and other negative developmental outcomes are well-documented (Clerkin et al., 2007; Thomas and Zimmer-Gembeck, 2007; Slobodskaya and Akhmetova, 2010; MacKenzie et al., 2012), the role of punitive parenting in child-well-being needs to be investigated in more detail.

### Interactive Effects

In this study, we investigated interactions between child personality and parenting practices in relation to child well-being. An interesting and important finding is that the effects of inconsistent parenting and corporal punishment on child well-being did not depend on child personality, whereas the effects of positive and punitive parenting on child well-being were moderated by personality traits. Future research should address multiple interactions among personality traits and more differentiated parenting practices to clarify the nature of this phenomenon.

Most of the interactions in our study supported a diathesis–stress model. Negative parenting practices strengthened the role of certain personality traits in predisposing children to low well-being, whereas positive parenting/involvement buffered this relation (Kiff et al., 2011; Slagt et al., 2016b; Stoltz et al., 2017). Put another way, certain personality traits amplified the strength of the association between negative parenting and children's problem behaviors and increased the protective role of positive parenting. Thus, punitive parenting was associated with externalizing problems among less conscientious and more physically active children; it was associated with internalizing problems among less extraverted and more fearful children, and with the impact of problems on everyday life among children higher in negative affect.

These findings are consistent with previous studies showing that the interaction of negative parenting with child temperament/personality traits generally follows a diathesis–stress pattern (Kiff et al., 2011; Meunier et al., 2011; Tung et al., 2019). Thus, children with higher impulsivity and irritability, lower effortful control, difficult temperaments or children higher in negative affectivity had a greater risk for adjustment problems in the context of negative parenting (Leve et al., 2005). Other studies have found that harsh parental discipline with corporal punishment was stronger related to externalizing problems among children lower in flexibility and higher in fussiness or negative affectivity (Kiff et al., 2011; Stoltz et al., 2017).

In one longitudinal study, boys with higher levels of fear/shyness at age 5, who experienced harsh discipline, had higher levels of internalizing problems at age 17 (Leve et al., 2005). A Dutch study of preschool children demonstrated that parental negative control strengthened the relation between fearfulness and internalizing problems (Karreman et al., 2010). If high fearfulness reflects a highly sensitive nervous system, then fearful children may experience more stress and anxiety in response to harsh discipline and punishment, and that may manifest as a high level of internalizing problems. Notably, one recent study provided evidence for the differential susceptibility model, showing that highly sensitive children had relatively more internalizing problems in the context of parental discord, but fewer problems when the relationship between parents was harmonious (Brock et al., 2017). In contrast to these findings, our results on the role of fearfulness in internalizing problems support the diathesis–stress model.

However, the results of the present study found support for the differential susceptibility to positive parenting/involvement among children with low levels of Beta superfactor, also called plasticity (DeYoung, 2006) or personal growth (Digman, 1997). Compared with their peers who were higher in plasticity, these children showed both more externalizing and more internalizing problems when they received less positive parenting, but significantly lower levels of total difficulties in the context of high-quality parenting. To our knowledge, this is the first study to demonstrate the differential susceptibility pattern of interaction effects at the higher-order level of personality traits. Beta higher-order factor includes biologically based temperamental dimension of surgency/positive emotionality, reflects the tendency to explore both behaviorally and cognitively (Rothbart, 2011; Shiner and DeYoung, 2013), and in some aspects resembles developmental plasticity as elaborated by Belsky and Pluess (2009).

Previous studies have established that children with difficult temperaments and children high in negative emotionality are more susceptible to childcare quality and to parental sensitivity and responsiveness (Pluess and Belsky, 2010). However, there is no evidence that difficult temperament or negative emotionality are the most important phenotypic markers of susceptibility. Identification of other potential markers thus remains a key issue and warrants further research (Belsky and Pluess, 2009; Pluess and Belsky, 2010). Our findings suggest that personality higher-order trait of Beta or plasticity comprising extraversion and openness may serve as a marker for susceptibility to environmental conditions. In a recent adult study of sensitivity groups, highly sensitive individuals were found to be high in neuroticism and low in extraversion (Lionetti et al., 2018). In the Lionetti's (2018) study, openness was not correlated with the total score on the highly sensitive person scale; it was positively correlated with the aesthetic sensitivity subscale and negatively correlated with two other subscales, ease of excitation, and low sensory threshold. There is also some evidence from adult studies suggesting that a temperamental trait of orienting sensitivity, which includes automatic cognitive activity, perceptual sensitivity, and affective sensitivity, could be an attentional substrate for the personality trait of openness (Evans and Rothbart, 2007).

Taken together, the current findings and those of other studies suggest that considering the role of temperament and personality traits at different levels of their hierarchical structure and at different developmental stages may provide important insights into the nature of trait-by-environment interactions. The results of the current study have major practical implications for interventions aimed to prevent or reduce child problem behavior. Our findings suggest that community-based interventions for parents aimed to improve developmental outcomes for young children by making their parenting more positive and responsive and less negative should take into account the individual differences of the child. These programs may reduce negative outcomes for children low in conscientiousness and extraversion or high in activity and fearfulness, whereas children low in Beta might benefit most from such intervention.

## Strengths and Limitations

There are some important strengths of the current study. First, regression analysis at three levels of personality hierarchy made it possible to replicate moderation effects at different levels of the hierarchy. Second, to our knowledge, no previously published studies have reported that personality characteristics may serve as phenotypic markers of differential susceptibility to parenting in relation to child well-being. Third, this study used a community sample of preschool children from diverse socioeconomic backgrounds, covering a full range of environments and developmental outcomes. Finally, while many studies examining environmental sensitivity relied on visual inspection of the interaction plots, this study used the regions of significance statistical analysis. The findings from this study contribute to the differential susceptibility theory and enhance our understanding of the mutually influential role of child personality and parenting behavior in child well-being.

However, the study also has some limitations that may restrict interpretation of the data. First, we had only one source of information—primary caregivers. Although parent reports do offer developmentally appropriate ratings with superior predictive validity with regard to other approaches, such as structured observations (Pauli-Pott et al., 2003), future research would benefit from the use of multiple informants along with observation-based measures. Second, the cross-sectional design gives no clear indication of causal influences; the findings should be confirmed by longitudinal study. Third, a relatively smaller sample size limited statistical power to detect significant interaction effects. Finally, this study was not genetically sensitive, therefore we cannot rule out influences of shared genes. Nevertheless, taking into account child personality traits, it was possible to obtain more robust associations of parenting with child well-being.

## Conclusion

The findings from this study enhance our understanding of the mutually influential role of child personality and parenting behavior in child well-being and the processes underlying diathesis–stress and differential susceptibility patterns. Thus, the effects of inconsistent parenting and corporal punishment on child well-being did not depend on child personality, whereas the effects of positive and punitive parenting on child well-being were moderated by personality traits. In line with a diathesis–stress framework, punitive parenting was a risk factor for externalizing problems among less conscientious and more physically active children and also a risk factor for internalizing problems among introverted and fearful children. Positive parenting/involvement was a protective factor for internalizing problems only for children low in openness. In line with a differential susceptibility framework, children low in Beta higher-order personality trait (also known as plasticity or personal growth) compared to high-plasticity peers showed more total problems when faced with low positive parenting, but fewer problems when experiencing high-quality parenting. To our knowledge, this is the first study to show that the higher-order personality trait may serve as a marker of differential susceptibility to parenting.

Research identifying the complex ways in which environmental factors and individual characteristics of children contribute to child well-being will confidently yield an increasing base of information that can be used to create prevention and intervention programs for children and their families. Thus, our findings have major practical implications suggesting that interventions aimed to prevent or reduce child problem behavior in preschool age by making their parenting more positive and consistent and less negative should take into account child personality traits, especially their level of plasticity, conscientiousness, extraversion, activity, and fearfulness.

## Data Availability Statement

The raw data supporting the conclusions of this article will be made available by the authors, without undue reservation.

## Ethics Statement

The studies involving human participants were reviewed and approved by State Research Institute of Physiology and Basic Medicine. The patients/participants provided their written informed consent to participate in this study.

## Author Contributions

SL contributed to the collection, analysis and interpretation of data, and drafting the article. HS contributed to the conception, design of the study, and revising the article critically for important intellectual content. All authors contributed to the article and approved the submitted version.

## Conflict of Interest

The authors declare that the research was conducted in the absence of any commercial or financial relationships that could be construed as a potential conflict of interest.

## Publisher's Note

All claims expressed in this article are solely those of the authors and do not necessarily represent those of their affiliated organizations, or those of the publisher, the editors and the reviewers. Any product that may be evaluated in this article, or claim that may be made by its manufacturer, is not guaranteed or endorsed by the publisher.
